# Analysis of mortality from suicide in the Altai Republic, Russia, for the period of 1990-2019

**DOI:** 10.1192/j.eurpsy.2022.1514

**Published:** 2022-09-01

**Authors:** N. Semenova

**Affiliations:** Scientific Research Institute of Medical Problems of the North, Department Of Child’s Physical And Mental Health, Krasnoyarsk, Russian Federation

**Keywords:** indigenous peoples, Altai Republic, Suicide, Epidemiology

## Abstract

**Introduction:**

The Altai Republic (AR) is a national republic of the Russian Federation (RF), where the indigenous people – Altaians live, and where a high death rate from suicide is recorded.

**Objectives:**

To analyze the dynamics of mortality from suicide in the AR for the period from 1990 to 2019.

**Methods:**

Data on mortality of the population were obtained from the Russian databases of demographic indicators. The data were analyzed in terms of indicators standardized per 100,000 population.

**Results:**

In the AR, as well as in the RF as a whole, there has been an increase in the level of suicide since 1990, but in Altai it lasted for a longer period of time – for 13 years. Throughout the entire period, the suicide rates in the AR have consistently exceeded the all-Russian indicators by 1.5-3 times, and by 2019 the gap in indicators has doubled compared to 1990. The curve of mortality from suicide in the AR has a fluctuating character with spontaneous peaks, in contrast to the curve in the RF that has the form of a “plateau”.

**Figure:**
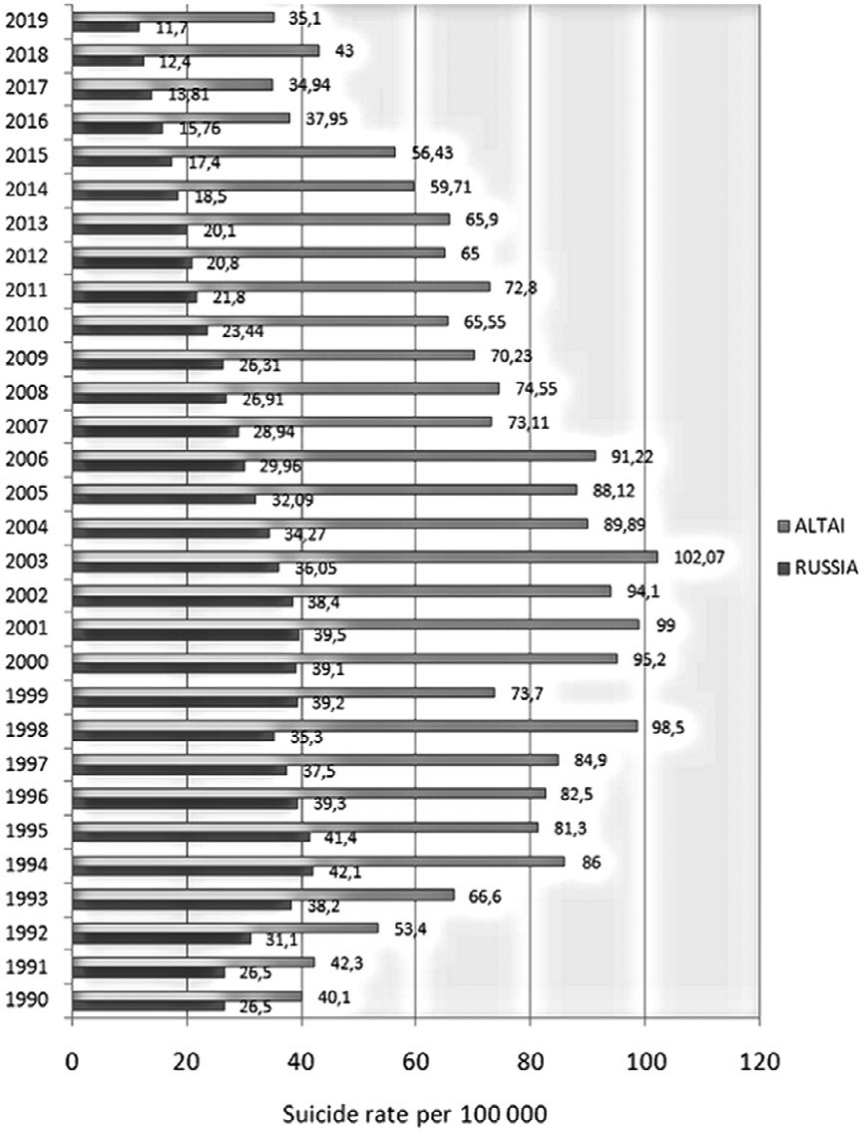

**Conclusions:**

In the AR, the mortality rate from suicide consistently exceeds the all-Russian indicators, by 2019 the gap in indicators has doubled. The mortality curve is fluctuating, which we associate with clustering of suicides. This phenomenon requires further study.

**Disclosure:**

No significant relationships.

